# A Hemodynamic Echocardiographic Approach to Disease Severity and Prognosis in Transthyretin Amyloid Cardiomyopathy

**DOI:** 10.1016/j.jacadv.2026.103244

**Published:** 2026-09-30

**Authors:** Matteo Serenelli, Anna Cantone, Aldostefano Porcari, Marco Pozzan, Marco Merlo, Gianfranco Sinagra, Gabriele Guidi Colombi, Beatrice Dal Passo, Federico Sanguettoli, Matteo Arzenton, Daniele Maio, Martina Vitali, Giulia Casolari, Jacopo Bonini, Alberto Cipriani, Laura De Michieli, Donato Mele, Giulio Sinigiani, Daniela Tomasoni, Marco Metra, Rita Pavasini, Simone Biscaglia, Gianluca Campo

**Affiliations:** a Cardiology Unit, Azienda Ospedaliera Universitaria di Ferrara, Ferrara, Italy; b Centre for Diagnosis and Treatment of Cardiomyopathies, Cardiovascular Department, Azienda Sanitaria Universitaria Giuliano-Isontina (ASUGI), University of Trieste, Trieste, Italy; c European Reference Network for Rare, Low Prevalence and Complex Diseases of the Heart-ERN GUARD-Heart; d National Amyloidosis Centre, Division of Medicine, University College London, Royal Free Hospital, London, United Kingdom; e Department of Cardio-Thoraco-Vascular Sciences and Public Health, University of Padua, Padua, Italy; f Cardiology Unit, University Hospital of Padua, Padua, Italy; g Cardiology, ASST Spedali Civili and Department of Medical and Surgical Specialties, Radiological Sciences, and Public Health, University of Brescia, Brescia, Italy

**Keywords:** amyloidosis, hemodynamic, prognosis, pulmonary artery pressure, stroke volume index, transthyretin

## Abstract

**Background:**

Transthyretin amyloid cardiomyopathy (ATTR-CM) causes heart failure and poor outcomes. Prognostic stratification is fundamental, yet validated staging systems rely exclusively on biomarkers, neglecting structural and functional cardiac parameters.

**Objectives:**

This study evaluates a noninvasive hemodynamic echocardiographic approach to stratify patients with ATTR-CM.

**Methods:**

Patients were categorized into 3 groups based on stroke volume index (SVi) and systolic pulmonary artery pressure (sPAP), markers of forward flow and elevated filling pressures. We compared receiver operating characteristic curves for various potentially predictive hemodynamic parameters to identify these as the most robust candidates for risk stratification. Optimal cutoffs (30 mL/m^2^ for SVi and 40 mm Hg for sPAP) were determined using the Liu index, supported by both univariable and multivariable survival analyses. The primary endpoint was the composite of all-cause mortality and heart failure hospitalization. Prognostic utility was evaluated using multivariable Cox regression, adjusted for age, sex, and disease-modifying therapy.

**Results:**

The cohort included 293 patients with ATTR-CM: group 1 (normal SVi/sPAP, n = 115 [39%]), group 2 (low SVi or high sPAP, n = 129), and group 3 (low SVi/high sPAP, n = 49). The primary outcome occurred in 108 (37%) patients. The multivariable analysis confirmed the staging system as an independent predictor of the composite outcome (group 2 HR: 1.70, 95% CI: 1.05-2.71; group 3 HR: 2.50, 95% CI: 1.44-4.30; *P* < 0.001).

**Conclusions:**

The noninvasive hemodynamic approach provides an alternative and promising method to stratify the risk of clinical outcome in patients with ATTR-CM, potentially improving management strategies.

Transthyretin amyloid cardiomyopathy (ATTR-CM) represents a challenging clinical entity characterized by the deposition of cleaved or misfolded transthyretin protein within the myocardium, leading to progressive heart failure (HF) and poor outcomes. Several risk-prediction models based on serum biomarkers have been developed for prognostic stratification in ATTR-CM to guide clinical decision-making and optimize patient care. The National Amyloidosis Centre (NAC) staging system, based on N-terminal pro-brain natriuretic peptide (≤3,000 or >3,000 ng/L) and estimated glomerular filtration rate (≥45 or <45 mL/min/1.73 m^2^), is among the most commonly used staging systems in clinical practice for patients with both wild-type transthyretin cardiac amyloidosis and variant transthyretin cardiac amyloidosis (ATTRv-CM).^1^ While biomarker-based staging systems are useful tools for prognostic stratification in ATTR-CM, they may not fully capture the heterogeneous natural history of ATTR-CM, which depends on multiple factors, including the severity of cardiac structural and functional amyloid-related abnormalities.

This study proposes a noninvasive hemodynamic echocardiographic stratification of ATTR-CM. Starting from the assumption that the leading causes of symptoms and decompensation in patients with HF with a preserved ejection fraction are impaired cardiac output reserve and chronically elevated left ventricular (LV) filling pressures, we postulate that a noninvasive estimation of hemodynamic parameters such as stroke volume index (SVi) and systolic pulmonary artery pressure (sPAP), will give an objective evaluation of cardiac status and presumably an accurate prediction of clinical outcomes.^2^

## Methods

### Study design

This is a multicenter, longitudinal, observational study performed across 4 referral centers for amyloid cardiomyopathy: Ferrara (Cardiovascular Unit, Ferrara University Hospital), Trieste (Cardiovascular Department, Azienda Sanitaria Universitaria Giuliano-Isontina), Brescia (Cardiovascular Unit, ASST Spedali Civili, University of Brescia), and Padua (Department of Cardiac, Thoracic and Vascular Sciences, University of Padova). Data of consecutive patients diagnosed with ATTR-CM between 2017 and 2023 were analyzed. The study was conducted according to the Declaration of Helsinki, and informed consent was obtained under the institutional review board policies of the relevant hospital administrations. Each participating center maintains a dedicated database where patient data are prospectively collected for clinical purposes.

Diagnosis of ATTR-CM was established either noninvasively or by tissue biopsy with confirmation of transthyretin amyloid deposit, as recommended by the international guidelines and consensus document.^3^^,^^4^ The nonbiopsy diagnosis was established based on HF symptoms together with a characteristic echocardiogram or cardiac magnetic resonance study and Perugini grade 2 or 3 myocardial uptake on cardiac scintigraphy with bone tracers confirmed by single-photon emission computerized tomography, in the absence of either an abnormal serum free light chain ratio or a monoclonal immunoglobulin in the serum or urine by immunofixation.^3^^,^^4^ The TTR gene was sequenced in all patients.

Patients were included in the study population only if data on SVi and sPAP were available at the echocardiographic assessment performed at ATTR-CM diagnosis.

Data regarding demographics, clinical characteristics, serum biomarkers, and transthoracic echocardiography were collected at the time of diagnosis. All patients were enrolled into a protocolized follow-up at participating centers with clinical assessment, laboratory tests, and echocardiography performed every 6 months. Participating centers shared an assessment protocol for follow-up, which aligned with the recommendations set forth in the European Society of Cardiology guidelines.^4^^,^^5^

### Echocardiographic assessment

Expert physicians with special competence in cardiomyopathy and cardiac amyloidosis performed all the echocardiographic examinations and measurements. Cardiac chamber dimensions, indexes of LV filling pressure, stroke volume (SV), and sPAP were calculated following the American Society of Echocardiography guidelines and the European Association of Cardiovascular Imaging.^6^^,^^7^

LV SV was calculated as the product of the left ventricular outflow tract (LVOT) area and the time-velocity integral of aortic flow velocity. The LVOT time-velocity integral was assessed by placing the pulsed-wave Doppler sample volume 0.5 to 1.0 cm proximal to the aortic valve in the apical five-chamber view. Measurements were averaged over 3 beats for patients in sinus rhythm and over 5 beats for those in atrial fibrillation. The diameter of the LVOT was measured in the parasternal long-axis view. SV was indexed to the body surface area to obtain the SVi. sPAP estimation was performed in accordance with the American Society of Echocardiography guidelines using the formula: sPAP = 4 × (vTR)^2^ + RAP, where RAP was assigned as 3, 8, or 15 mm Hg based on IVC diameter (≤2.1 or >2.1 cm) and inspiratory collapse (≥50% or <50%, respectively).

Patients were classified into 3 groups according to SVi (>30 or ≤30 mL/m^2^) and sPAP (≥40 or <40 mm Hg). These cutoffs were chosen based on logistic and Cox regression analysis results. The cutoff of 30 mL/m^2^ was also in line with previous evidence in patients with HF.^8^ Patients were therefore classified into group 1 (low risk): SVi >30 mL/m^2^ and sPAP <40 mm Hg; group 2 (moderate risk) SVi ≤30 mL/m^2^ or sPAP ≥40 mm Hg; and group 3 (high risk) SVi ≤30 mL/m^2^ and sPAP ≥40 mm Hg.

### Outcomes

The composite primary outcome was the occurrence of all-cause death and HF hospitalization. The mortality endpoint was defined as time to death from baseline for all deceased patients and time to censor date from baseline among the remainder. The secondary endpoint was the occurrence of all-cause death or HF hospitalization evaluated individually. Clinical events were determined by systematic electronic record review and confirmed by expert investigators at each site.

### Statistical analyses

Continuous data were expressed as mean ± SD if they presented a normal distribution or as median and interquartile range otherwise. Categorical data were expressed as numbers and percentages and compared using the Pearson chi-square test or Fisher exact test. Ordinal variables across categories were compared using the Mantel-Haenszel chi-square test for trend in ordinal variables and ANOVA/Kruskal-Wallis for continuous data; analysis was performed using the Student *t*-test or Mann-Whitney test if non-normally distributed. A *P* for trend was also provided based on hemodynamic groups.

To define the staging system, optimal cut-points for relevant variables (sPAP and SVi) were initially determined using receiver operating characteristic curve analysis and the Liu index method (Supplemental Figures 1 and 2 in Supplemental Appendix). The Liu index is derived from a nonparametric approach and defines the optimal threshold by maximizing the product of sensitivity and specificity, thereby providing a mathematically balanced trade-off between true-positive and true-negative rates.

These cut-points were both good discriminators of survival by log-rank tests (Supplemental Figures 3 and 4 in Supplemental Appendix). Based on the results of the Liu method, different SVi (25, 30, or 35 mL/m^2^) and sPAP cutoffs (30, 35, or 40 mm Hg) were also tested for their discriminatory ability and proportional representation in a multivariable Cox regression model. Harrell’s c-statistic was calculated to measure the discriminatory ability of each model. The SVi cut-point of 30 mL/m^2^ when combined with the sPAP cut-point of 40 mm Hg showed the best Harrell’s C while dividing an acceptable proportion of patients into each of the 3 stages.

Cox models were tested to account for potential nonproportional hazards over time with Schoenfeld residuals. Survival was evaluated using a multivariable Cox proportional hazard regression analysis, providing estimated HRs with 95% CIs. To account for different time exposure to disease-modifying treatment (DMT), cumulative days on treatment were modelled as a time-dependent covariate during follow-up.

The Kaplan-Meier method was used to estimate the probability of remaining free from the study endpoint at any given time point during follow-up, and the log-rank test was used to compare the curves.

For the endpoint of HF hospitalization, we used a competing-risk framework based on the Fine-Gray subdistribution hazard model to estimate subdistribution HRs (sHR), with all-cause mortality treated as a competing event. Cumulative incidence functions were calculated for each group and graphically displayed to compare the incidence of HF hospitalization while accounting for the competing risk of death. To correct optimism, internal validation was performed for prediction models using a bootstrap procedure with 1,000 bootstrapped samples.

Reproducibility was evaluated in a randomly selected sample of 30 patients (10% of the cohort). Two independent observers, blinded to each other's measurements and to clinical outcomes, performed the measurements for the inter-rater analysis. For the intra-rater analysis, one observer repeated the measurements after an appropriate time interval while blinded to the initial measurements. Intraclass correlation coefficients were estimated using a two-way mixed-effects model with single measurements, and 95% confidence intervals were calculated (Supplemental Table 1 in Supplemental Appendix).

All statistical analyses were performed using STATA version 16 (Stata Corp LLC) and R (R Foundation for Statistical Computing). A *P* value <0.05 was considered statistically significant. The corresponding author had full access to all the data in the study, taking responsibility for the integrity of the data and accuracy of the data analysis.

## Results

### Patient population

Out of 396 patients initially screened, inferior vena cava measurements were not feasible in 3% of cases, whereas peak tricuspid regurgitation velocity via continuous-wave Doppler could not be obtained in 26% of patients because of the absence of a sufficient tricuspid regurgitation jet or an inadequate ultrasound window. A total of 293 patients were enrolled in the analysis. The study cohort had a mean age of 78.6 ± 8.9 years, 21.2% women, 91.1% wild-type transthyretin cardiac amyloidosis, and 8.9% ATTRv-CM. Among the 26 patients with ATTRv-CM, one had p.Val142Ile-associated ATTRv-CM, and 25 (96.1%) had non-p.Val142Ile/non-p.Thr80Ala-associated ATTRv-CM (p.Ile88Leu, 17 patients; p.Val50Met, 2 patients; and one patient each with p.Glu54Gln, p.Glu82Lys, p.Tyr78Phe, p.Glu109Gln, p.Val114Leu, and p.Val94Leu, respectively).

### Hemodynamic categorization in relation to baseline characteristics and symptoms

Table 1 shows baseline characteristics of patients with ATTR-CM according to the hemodynamic groups: 115 patients (39%) were in group 1 (low risk), 129 (44%) in group 2 (intermediate risk), and 49 (17%) in group 3 (high risk).

**Table 1:** Baseline Clinical, Biomarker, and Echocardiographic Characteristics Stratified by Hemodynamic Group

	Total (N = 293)	Group 1 (n = 115)	Group 2 (n = 129)	Group 3 (n = 49)	*P* Value for Trend
Age, y	78.6 (8.9)	78.0 (7.6)	79.3 (10.8)	78.5 (6.0)	0.53 0.98
Female, n (%)	62 (21.2)	20 (17.4)	34 (26.4)	8 (16.3)	0.15 0.96
ATTRv-CM, n (%)	26 (8.9)	13 (11.3)	7 (5.4)	6 (12.2)	0.18 0.92
SBP (mm Hg)	127.7 (17.9)	127.3 (17.8)	127.8 (18.7)	128.5 (16.5)	0.091 0.65
Heart rate (beats/min)	73.6 (15.4)	70.3 (11.7)	74.9 (17.5)	78.0 (15.8)	0.005 0.003
AF/AFL at basal ECG	117 (40)	27 (23)	66 (51)	24 (49)	<0.001 0.06
NYHA functional class					<0.001 <0.001
I	69 (23.6)	38 (33.0)	25 (19.5)	6 (12.2)	
II	144 (49.3)	63 (54.8)	64 (50.0)	17 (34.7)	
III	76 (26.0)	14 (12.2)	37 (28.9)	25 (51.0)	
IV	3 (1.3)	0 (0.0)	3 (2.3)	1 (2.0)	
NAC ATTR-CM stage					<0.001 <0.001
I	168 (57.3)	85 (73.9)	64 (49.6)	19 (38.8)	
II	83 (28.3)	21 (18.3)	43 (33.3)	19 (38.8)	
III	42 (14.3)	9 (7.8)	22 (17.1)	11 (22.4)	
Echocardiographic parameters					
EDVi (mL/m^2^)	52.4 (14.8)	52.7 (13.1)	51.3 (16.0)	54.9 (15.7)	0.34 0.71
IVSd (mm)	17.1 (3.2)	16.5 (2.8)	17.5 (3.5)	17.6 (2.7)	0.034 0.025
PWd (mm)	15.3 (3.1)	14.8 (3.2)	15.6 (3.0)	15.4 (3.0)	0.16 0.17
LV mass index (g/m^2^)	166.9 (49.5)	154.8 (48.2)	172.9 (51.4)	178.6 (42.0)	0.003 0.002
GLS (%)	−11.0 (5.5)	−12.1 (6.5)	−10.9 (4.1)	−8.6 (5.6)	0.004 <0.001
LVEF (%)	51.6 (10.9)	56.0 (8.0)	51.1 (10.9)	42.6 (10.8)	<0.001 <0.001
SVi (mL/m^2^)	31.9 (9.5)	38.2 (7.9)	29.8 (8.4)	22.6 (4.9)	<0.001 <0.001
sPAP (mm Hg)	37.7 (11.7)	29.3 (6.6)	40.7 (11.4)	49.5 (6.7)	<0.001 <0.001
E/A ratio	2.0 (1.1)	1.7 (1.0)	2.4 (1.1)	2.5 (1.0)	<0.001 <0.001
E/e’ ratio	17.6 (7.1)	15.2 (5.6)	19.3 (7.5)	18.7 (7.9)	0.001 0.003
Lateral S’-wave (cm/s)	5.5 (1.8)	6.0 (1.9)	5.3 (1.6)	4.7 (1.3)	0.001 <0.001
Beta-blockers, n (%)	160 (55.4)	48 (42.1)	82 (64.1)	30 (63.8)	0.003 0.008
Loop diuretics dose (mg)	58.4 (109.3)	41.9 (115.2)	56.3 (91.4)	102.7 (127.1)	<0.001 <0.001
Disease-modifying treatment	123 (42.0)	60 (52.2)	50 (38.8)	13 (26.5)	0.006 0.002

AF/AFL = atrial fibrillation/atrial flutter; ATTRv-CM = variant transthyretin cardiac amyloidosis; ECG = electrocardiography; EDVi = end-diastolic volume index; GLS = global longitudinal strain; IVSd = interventricular septal thickness in diastole; LVEF = left ventricular ejection fraction; LV mass index = left ventricular mass index; NAC ATTR-CM stage = National Amyloidosis Centre staging for ATTR-CM; PWd = posterior wall thickness in diastole; SBP = systolic blood pressure; sPAP = systolic pulmonary artery pressure; SVi = stroke volume index; S’ = Systolic Mitral Annular Velocity (Tissue Doppler Imaging).

Patients in group 1 showed the lowest baseline heart rate while those in group 3 showed the highest (*P* trend 0.003). Furthermore, group 1 had the lowest rate of atrial fibrillation/flutter history (*P* < 0.001).

The distribution of NYHA functional class varied significantly across hemodynamic groups, with an increasing proportion of patients with NYHA class III and IV from group 1 (12.2%) to group 2 (30.5%) and 3 (53.0%) (*P* trend <0.001) (Table 1).

The distribution of NAC ATTR-CM stages also varied across the hemodynamic groups, with an increasing proportion of patients presenting with NAC ATTR stage III from group 1 (7.8%) to group 2 (17.1%), and group 3 (22.4%) (*P* trend <0.001). Median dose of loop diuretics was 41.9, 56.3, and 102.7 mg/day for group 1, 2, and 3, respectively (*P* trend <0.001). There was no difference among patients in different hemodynamic groups when considering age, sex, and proportion of patients with ATTRv-CM (all *P*/*P* trend >0.05).

### Hemodynamic categorization in relation to echocardiographic phenotype

Patients from group 1 to group 3 showed increasing values of LV mass index (154.8, 172.9, and 178.6 g/m^2^, *P* trend = 0.002) and decreasing values of LV global longitudinal strain (LV GLS) (−12.1%, −10.9%, and −8.6%, *P* trend <0.001), left ventricular ejection fraction (56.0%, 51.1%, and 42.6%, *P* trend <0.001), and lateral S’-wave (6.0, 5.3, and 4.7 cm/s, *P* trend <0.001). E/e’ was significantly lower in group 1 than in groups 2 and 3 (Table 1).

A total of 123 patients (42% of the cohort) were receiving DMT, with a significant downward trend in treatment rates from group 1 to group 3 (52.2%, 38.8%, and 26.5%, respectively; *P* trend <0.001). Among those on DMT, the most frequently prescribed agent was tafamidis (94%), followed by vutrisiran (4%) and eplontersen (2%).

### Prognostic value of the hemodynamic classification

#### Composite of all-cause death and HF hospitalization

Over a median follow-up of 19.8 months (IQR: 13.0-31.0), 108 patients (37%) experienced the primary composite endpoint of all-cause death or HF hospitalization. Event rates progressively increased across hemodynamic groups: 23% in group 1, 41% in group 2, and 59% in group 3 (*P* < 0.001) (Table 2). The crude incidence rate per 100 person-years was 11.8 in group 1, 23.7 in group 2, and 38.3 in group 3 (*P* < 0.001).

**Table 2:** Event Incidence and Hazard Ratios According to Hemodynamic Risk Categories

	Death or Heart Failure Hospitalization			All-Cause Mortality			Heart Failure Hospitalization^a^		
	Group 1 Low Risk (n = 115)	Group 2 Intermediate Risk (n = 129)	Group 3 High Risk (n = 49)	Group 1 Low Risk (n = 115)	Group 2 Intermediate Risk (n = 129)	Group 3 High Risk (n = 49)	Group 1 Low Risk (n = 115)	Group 2 Intermediate Risk (n = 129)	Group 3 High Risk (n = 49)
Event number (%)	26 (23)	53 (41)	29 (59)	18 (16)	38 (29)	24 (49)	15 (13)	27 (21)	12 (24)
Rate per 100 person-year (95% CI)	11.8 (8.0-17.3)	23.7 (18.1-31.0)	38.3 (26.4-55.4)	7.2 (4.6-11.5)	14.6 (10.6-20.1)	29.8 (20.0-44.4)	6.8 (4.1-11.3)	12.0 (8.2-17.5)	16.7 (9.5-29.4)
HR (95% CI)	1.00, reference	1.96 (1.22-3.15)	3.14 (1.85-5.40)	1.00, reference	1.96 (1.12-3.44)	3.97 (2.14-7.38)	1.00, reference	2.08 (1.03-4.17)	3.82 (1.78-8.17)

a Heart failure hospitalization was evaluated using a Fine-Gray competing-risk approach, with death treated as a competing event to provide unbiased estimates of the subdistribution hazard.

At univariable Cox regression, the hemodynamic classification was significantly associated with the primary endpoint, with group 1 as reference (HR: 1.00), and increasing risks observed in group 2 (HR: 1.96; 95% CI: 1.22-3.15) and group 3 (HR: 3.14; 95% CI: 1.85-5.40; *P* < 0.001) (Figures 1A and 1B).

**Figure 1:**
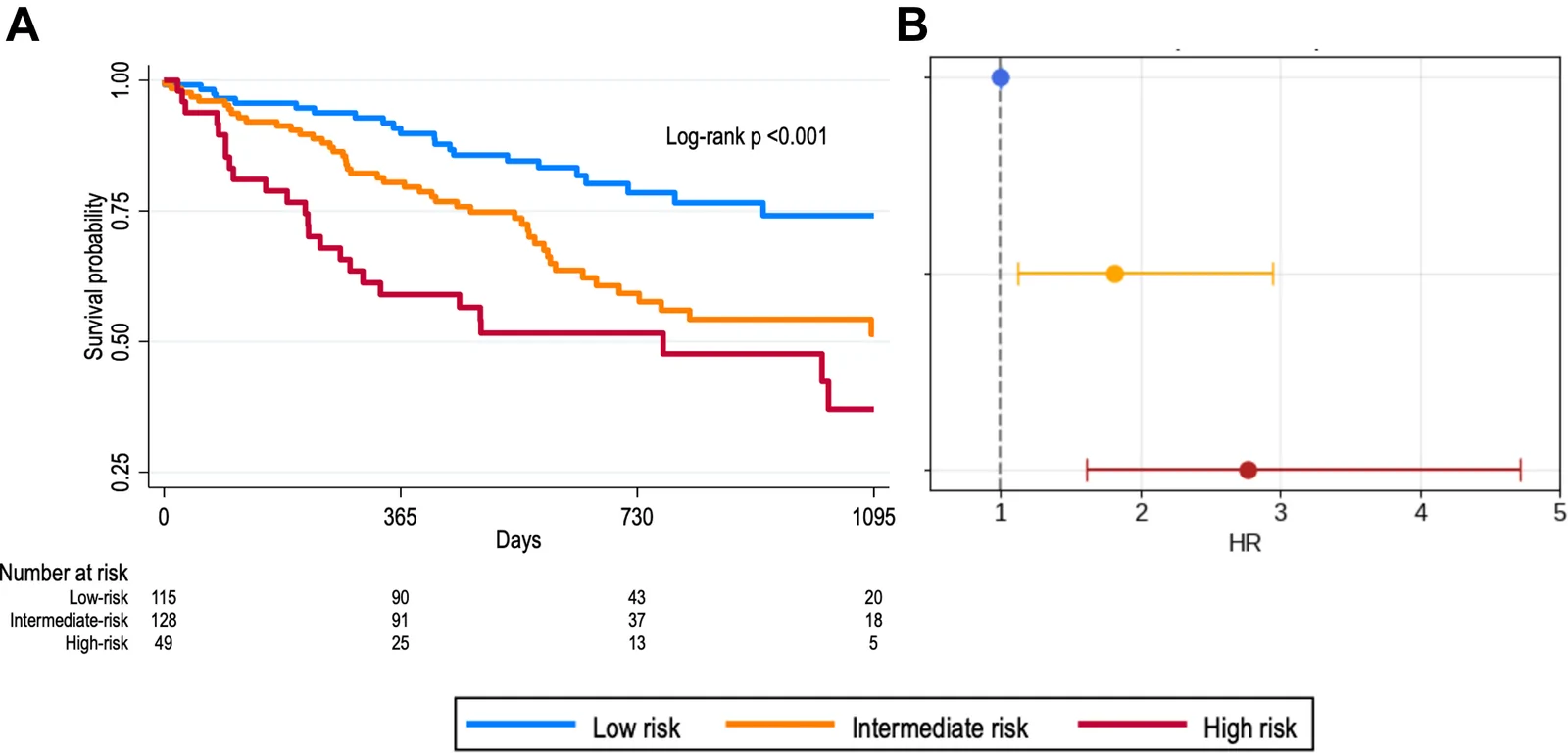
Kaplan-Meier Survival Curves (A) and HRs (B) for the Primary Composite Outcome Across Hemodynamic Groups

In the multivariable Cox model adjusted for age, sex, and time on disease-modifying therapy, the hemodynamic classification remained an independent predictor of outcome (HR: 1.70 and 95% CI: 1.05-2.71 for group 2 and HR: 2.50 and 95% CI: 1.44-4.30 for group 3; *P* < 0.001) (Table 3). The Harrell’s C-statistic for this model was 0.73. Internal validation using bootstrap resampling yielded an optimism-corrected C-statistic of 0.72. In the exploratory multivariable analyses adjusting for NAC stage, both hemodynamic classification and NAC stage remained independent predictors of the composite outcome (Supplemental Table 2 in Supplemental Appendix).

### All-cause mortality

A total of 80 patients (27.3%) died during follow-up, including 16.0% in group 1, 29% in group 2, and 49% in group 3 (*P* < 0.001) (Table 2). Crude mortality rates per 100 person-years were 7.2, 14.6, and 29.8 in groups 1, 2, and 3, respectively (*P* < 0.001).

The hemodynamic classification was strongly associated with all-cause mortality in univariable Cox regression: group 1 as reference (HR: 1.00), group 2 HR: 1.96 (95% CI: 1.12-3.44), and group 3 HR: 3.97 (95% CI: 2.14-7.38; *P* < 0.001) (Figure 2).

**Figure 2:**
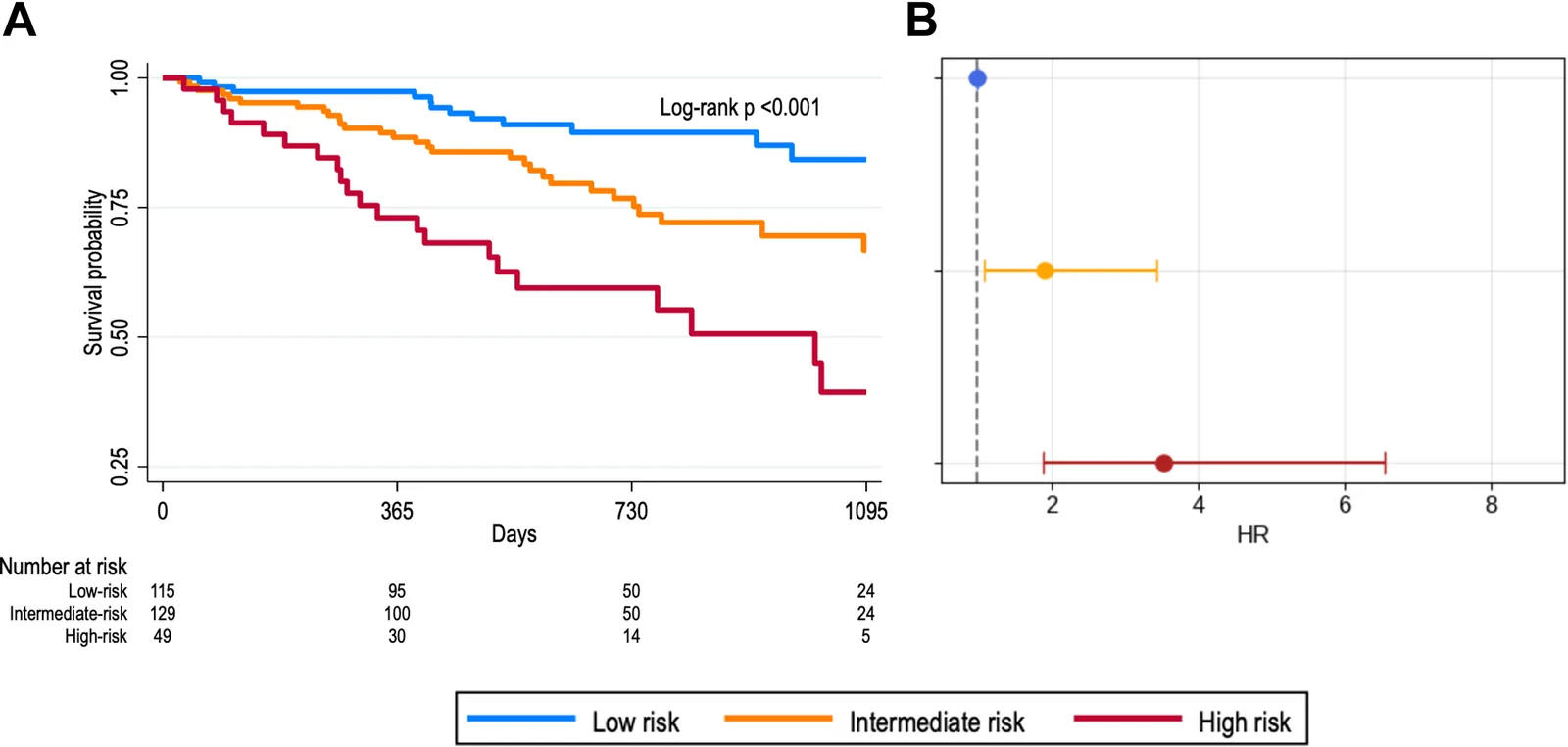
Kaplan-Meier Survival Curves (A) and HRs (B) for All-Cause Death Across Hemodynamic Groups

In the multivariable model adjusted for age, sex, and disease-modifying therapy, the classification remained independently predictive (HR: 1.68 and 95% CI: 1.02-2.79 for group 2 and HR: 3.16 and 95% CI: 1.70-5.87 for group 3; *P* < 0.001) (Table 3). The Harrell’s C-statistic was 0.74. In the exploratory multivariable analyses adjusting for NAC stage, both hemodynamic classification and NAC stage remained independent predictors of the all-cause mortality (Supplemental Table 2 in Supplemental Appendix).

**Table 3:** Multivariable Regression Models for Prediction of the Primary and Secondary Outcomes

	Death or HF Hospitalization			All-Cause Mortality			HF Hospitalization^a^		
	HR	95% CI	*P* Value	HR	95% CI	*P* Value	sHR^a^	95% CI	*P* Value
Hemodynamic group			<0.001			<0.001			<0.001
Group 1, low risk	1.00, reference	-		1.00, reference	-		1.00, reference	-	
Group 2, intermediate risk	1.70	1.05-2.71		1.68	1.02-2.79		1.81	0.91-3.60	
Group 3, high risk	2.50	1.44-4.30		3.16	1.70-5.87		2.97	1.38-6.35	
Age (1-y increase)	1.03	0.99-1.07	0.20	1.04	1.00-1.08	0.045	1.02	0.99-1.05	0.17
Female	0.66	0.31-1.37	0.26	0.49	0.26-0.95	0.033	0.57	0.33-0.98	0.041
Days on disease-modifying treatment	1.00	0.99-1.00	<0.001	1.00	0.99-1.00	<0.001	1.00	1.00-1.00	0.045
ATTRv-CM	0.97	0.40-2.33	0.95	1.19	0.40-3.60	0.74	1.44	0.47-4.37	0.52
	Harrel’s C = 0.73			Harrel’s C = 0.74			Not applicable		

ATTRv-CM = variant transthyretin cardiac amyloidosis; HF = heart failure; sHR = subdistribution HRs.

a Heart failure hospitalization was evaluated using a Fine-Gray competing-risk approach, with death treated as a competing event to provide unbiased estimates of the subdistribution hazard.

### HF hospitalization

HF hospitalization occurred in 55 patients (18.8%): 13% in group 1, 21% in group 2, and 24% in group 3. The crude incidence rate per 100 person-years was 6.8 in group 1, 12.0 in group 2, and 16.7 in group 3 (*P* < 0.001).

At univariable competing-risk analysis, the hemodynamic classification was significantly associated with HF hospitalization: group 1 as reference (sHR: 1.00), group 2 sHR: 2.08 (95% CI: 1.03-4.17), and group 3 sHR: 3.82 (95% CI: 1.78-8.17; *P* = 0.003) (Figure 3).

**Figure 3:**
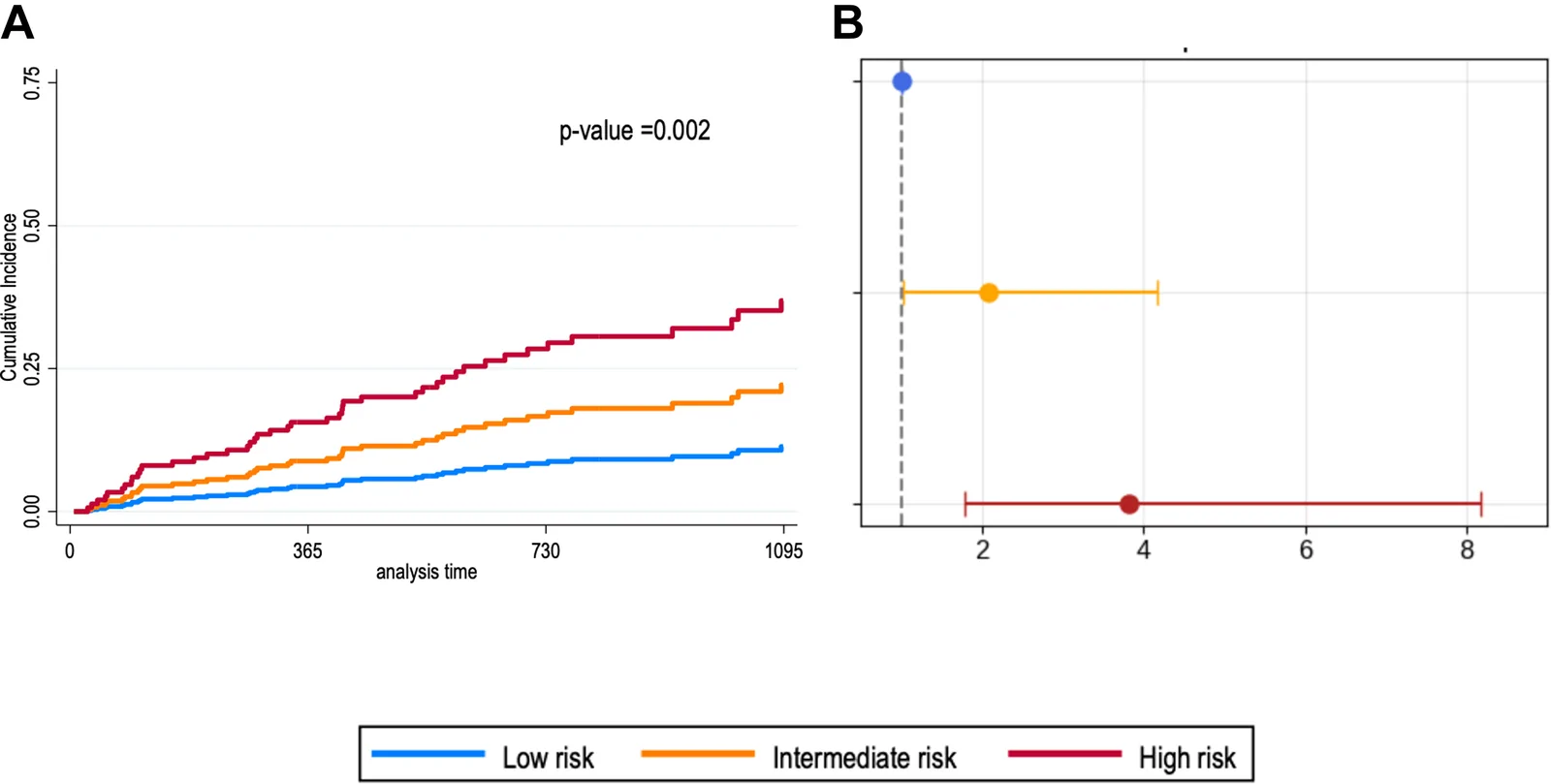
Cumulative Incidence Function Curves (A) and Subdistribution HRs (B) for Heart Failure Hospitalization across Hemodynamic Groups

**Central Illustration:**
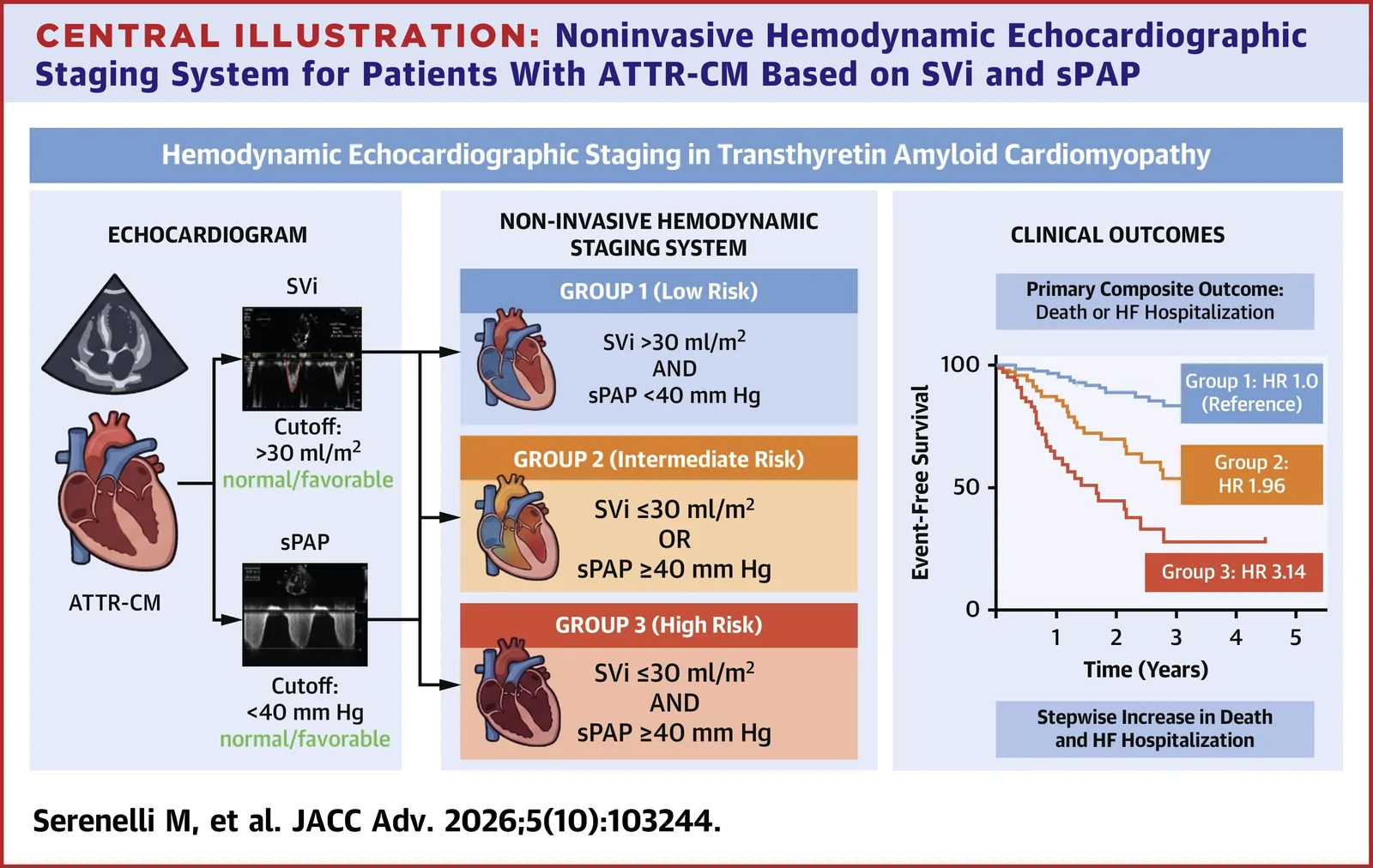
Noninvasive Hemodynamic Echocardiographic Staging System for Patients With ATTR-CM Based on SVi and sPAP Patients are stratified into 3 distinct risk categories (group 1: low risk; group 2: intermediate risk; group 3: high risk) based on values of SVi and sPAP. ATTR-CM = transthyretin amyloid cardiomyopathy; HF = heart failure; sPAP = systolic pulmonary artery pressure; SVi = stroke volume index.

In the multivariable competing-risk model adjusted for age, sex, and disease-modifying therapy, the classification remained an independent predictor, with sHR: 1.81 (95% CI: 0.91-3.60) for group 2 and sHR: 2.97 (95% CI: 1.38-6.35) for group 3 (*P* = 0.019) (Table 3). In the exploratory multivariable analyses adjusting for NAC stage, hemodynamic classification remained an independent predictor of HF hospitalization (Supplemental Table 2 in Supplemental Appendix).

## Discussion

In this multicenter cohort of patients with ATTR-CM, we demonstrate that a simple, noninvasive hemodynamic stratification based on SVi and sPAP provides powerful and independent prognostic information (Central Illustration). This approach identified a clear stepwise increase in the risk of all-cause death and HF hospitalization, independent of age, sex, TTR genotype, and disease-modifying therapy, and it remained predictive when each component of the primary endpoint was evaluated separately. These findings support the concept that cardiac structural and functional impairment—rather than biomarkers alone—represents a critical determinant of the clinical trajectory in ATTR-CM.^8^, ^9^, ^10^

Prognostic assessment in ATTR-CM remains challenging given the heterogeneity of cardiac involvement and the limited ability of current staging models to quantify the key hemodynamic derangements underpinning symptom burden. Existing prognostic systems rely exclusively on circulating biomarkers (N-terminal pro-brain natriuretic peptide, troponin, and renal function), and although they have been widely validated, they do not directly capture the central pathophysiological features of restrictive and infiltrative cardiomyopathies: impairment of forward flow and chronically elevated filling pressures. Our findings indicate that the combination of two key hemodynamic hallmarks—reduced SVi and elevated sPAP—provides independent prognostic value in ATTR-CM, even after adjusting for NAC staging. This observation parallels the importance of cardiac output limitation and pulmonary hypertension in the development of congestion, decompensation, and mortality due to HF with a preserved ejection fraction and other restrictive cardiomyopathies.^8^ The rationale for using these measures stems from the need to evaluate parameters directly associated with cardiac morbidity, rather than surrogate biomarkers. SVi is a comprehensive measure of ventricular performance, integrating preload, afterload, contractility, chamber geometry, and mitral regurgitation, and has been shown to decline early in cardiac amyloidosis.^10^, ^11^, ^12^, ^13^ Conversely, sPAP incorporates information from both left- and right-sided function and reflects elevated LV filling pressures. Furthermore, while indexes of diastolic properties (ie, E/e′ ratio) and myocardial deformation (ie, GLS) hold well-established prognostic value, our intention was to focus on SVi and sPAP as key hemodynamic markers of disease progression. Pathophysiologically, severe diastolic dysfunction and increased ventricular stiffness are among the primary drivers of impaired filling. This impairment leads to a downstream reduction in SV and a backward transmission of pressures into the pulmonary circulation. SVi and sPAP act as the final common pathway and clinical effectors of advanced diastolic and systolic impairment in this cohort, which led to their selection as the primary metrics for our risk categorization. Specifically, sPAP was chosen as a marker of hemodynamic congestion because it comprehensively reflects the cumulative consequences of chronically elevated left-sided filling pressures, pulmonary vascular involvement, and right heart burden—all of which are highly relevant, downstream determinants of disease severity and prognosis in ATTR-CM. Only a few studies have examined hemodynamics in cardiac amyloidosis. Reduced cardiac index at catheterization was independently associated with outcomes across amyloid etiologies.^14^ However, an invasive hemodynamic assessment is limited to selected patients and is impractical for routine follow-up. More recently, Venneri et al^15^ demonstrated that a >5% reduction in LVOT time-velocity integral over 12 months, measured using an artificial intelligence–based analysis, was an independent predictor of all-cause mortality in a cohort of patients with ATTR-CM.

Our study applies a noninvasive hemodynamic approach feasible in a contemporary ATTR-CM population, demonstrating robust association with symptoms and excellent prognostic ability.

The progressive worsening of clinical and echocardiographic markers from group 1 to group 3—increasing LV mass, worsening GLS and left ventricular ejection fraction, higher NYHA functional class, and greater diuretic requirements—indicate that the proposed classification reflects true disease severity and systemic congestion rather than arbitrary clustering. Beyond the amyloid load, these hemodynamic changes likely drive end-organ dysfunction: Venous congestion exacerbates renal and hepatic hypoperfusion and becomes particularly detrimental in individuals with low SV. This hemodynamic stratification has several advantages for contemporary management of ATTR-CM. First, it is easily derived during a standard echocardiogram, without additional software, cost, or examination time. Second, it may complement biomarker-based staging systems by identifying a distinct dimension of risk. Third, in the era of increasing disease prevalence and growing amyloidosis clinics, it allows for pragmatic differentiation of follow-up intensity: Patients in higher-risk groups may require closer surveillance, prioritization for disease-modifying therapy, more aggressive diuretic adjustment, earlier HF referral, or advanced HF therapies.

Finally, because disease-modifying drugs have been shown to modify SVi trajectory, this noninvasive approach may also be applicable for treatment monitoring and longitudinal follow-up.

Notably, patients at the highest event risk (group 3) were less likely to receive disease-modifying therapy than those in lower-risk groups. This observation is seemingly paradoxical, given that these patients—being the most hemodynamically compromised—would arguably derive the greatest benefit from aggressive therapeutic intervention. However, this discrepancy is largely driven by the current regulatory landscape: the Italian Medicines Agency restricts tafamidis reimbursement to patients in NYHA functional class I to II. Consequently, patients already in NYHA class III are legally ineligible for treatment, explaining the lower therapy rates observed in this advanced subgroup.

Although our hemodynamic classification captures key features of cardiac dysfunction, risk stratification could be further enhanced by incorporating supplementary metrics of right ventricular (RV) performance. Emerging evidence highlights the prognostic relevance of pulmonary hemodynamics, RV-pulmonary arterial coupling, and specific indexes of RV systolic function—such as TAPSE, RV S', and RV GLS—in patients with ATTR-CM.^16^^,^^17^ Although these parameters were not formally assessed in our cohort, their routine integration alongside sPAP may significantly refine prognostic accuracy in future clinical practice.

### Study Limitations

The primary limitation of this study is the absence of an external validation cohort, which substantially restricts the generalizability of our findings. Further prospective external validation is needed, ideally incorporating serial imaging to evaluate whether transitions between hemodynamic groups reflect disease progression or treatment response. Furthermore, integrating hemodynamic classification with biomarkers and strain-based metrics may yield an even more refined prognostic tool. Another potential limitation includes the lack of an independent event adjudication committee. Furthermore, sPAP estimation is fundamentally limited in patients with inadequate tricuspid regurgitation or suboptimal acoustic windows—which accounted for 26% of our cohort. In such cases, pulmonary artery acceleration time represents a valuable alternative metric to explore in future studies, with the clinical aim of improving feasibility and diagnostic yield.

Finally, the absence of a centralized core laboratory for echocardiographic analysis may have introduced some degree of interobserver variability. Although all measurements were performed by experienced operators following standardized guidelines—and the results of our interobserver and intraobserver reproducibility analyses demonstrated satisfactory agreement—this remains a potential limitation of our study.

## Conclusions

We propose a noninvasive, physiology-driven hemodynamic classification that provides prognostic value in ATTR-CM, predicts mortality and HF hospitalization beyond biomarkers alone, and may contribute to personalized follow-up strategies in contemporary clinical practice.

Perspectives **COMPETENCY IN PATIENT CARE AND PROCEDURAL SKILLS:** Noninvasive echocardiographic hemodynamic assessment integrating stroke volume index and systolic pulmonary artery pressure allows risk stratification of patients with transthyretin amyloid cardiomyopathy into three distinct prognostic groups. This bedside staging system captures forward-flow failure and pulmonary congestion, predicting all-cause mortality and heart failure hospitalization.

**TRANSLATIONAL OUTLOOK**: Incorporating routine Doppler-derived hemodynamics enables clinicians to identify patients at higher risk of clinical decline, tailor follow-up surveillance, and guide decongestive therapy titration.

## Funding support and author disclosures

The authors have reported that they have no relationships relevant to the contents of this paper to disclose.
