# Supplementary material for: A Hemodynamic Echocardiographic Approach to Disease Severity and Prognosis in Transthyretin Amyloid Cardiomyopathy
Source: JACC Adv. 2026 Sep 30;5(10):103244. doi: 10.1016/j.jacadv.2026.103244 (PMC13699706; doi:10.1016/j.jacadv.2026.103244)
Supplement: Supplementary file 1 [file mmc1.docx]

**SUPPLEMENTARY APPENDIX**

**Table S1. Assessment of Intra- and Inter- Rater Reproducibility.**

| **Echocardiographic Parameter** | **Intra-Rater ICC (95% CI)** | **Inter-Rater ICC (95% CI)** |
| --- | --- | --- |
| **Stroke Volume Index (ml/m^2^)** | 0.94 (0.87 - 0.97) | 0.86 (0.72 - 0.93) |
| **Systolic Pulmonary Artery Pressure (mmHg)** | 0.94 (0.88 - 0.97) | 0.90 (0.81 - 0.95) |

ICC stand for Interclass Correlation Coefficient.
**Table S2. Multivariable Regression Models for Prediction of the Primary and Secondary Outcomes adjusted for NAC stage**

| **Variables** | **Death or HF**  **hospitalization** | | | **All-Cause Mortality** | | | **HF hospitalization*** | | |
| --- | --- | --- | --- | --- | --- | --- | --- | --- | --- |
|  | **HR** | **95% CI** | **P-value** | **HR** | **95% CI** | **P-value** | **sHR*** | **95% CI** | **P-value** |
| **Hemodynamic group** |  |  | 0.041 |  |  | 0.021 |  |  | 0.040 |
| Group 1  Low Risk | 1-reference | - |  | 1-reference | - |  | 1-reference | - |  |
| Group 2  Intermediate Risk | 1.46 | 1.13–3.91 |  | 1.58 | 0.94–2.66 |  | 1.63 | 1.14–5.52 |  |
| Group 3  High Risk | 2.11 | 0.99–3.45 |  | 2.59 | 1.32-5.09 |  | 2.51 | 1.38–6.35 |  |
| **NAC stage** |  |  | <0.001 |  |  | <0.001 |  |  | 0.11 |
| I | 1-reference |  |  | 1-reference |  |  | 1-reference |  |  |
| II | 1.50 | 0.92-2.42 |  | 1.52 | 0.86-2.69 |  | 1.89 | 1.01-3.54 |  |
| III | 3.04 | 1.70-5.43 |  | 2.63 | 1.51-4.61 |  | 1.74 | 0.80-3.81 |  |
| **Age (1-year**  **increase)** | 1.01 | 0.99 - 1.03 | 0.40 | 1.04 | 0.99-1.08 | 0.054 | 1.02 | 0.98-1.06 | 0.26 |
| **Female sex** | 0.65 | 0.39 – 1.11 | 0.11 | 0.61 | 0.31 - 1.18 | 0.15 | 0.74 | 0.35-1.57 | 0.43 |
| **Days on**  **disease-modifying treatment** | 0.99 | 0.99 – 1.00 | <0.001 | 1.00 | 0.99 – 1.00 | <0.001 | 1.00 | 0.99 – 1.00 | <0.001 |
| **vTTR** | 0.70 | 0.28 – 1.75 | 0.45 | 0.98 | 0.35-2.73 | 0.97 | 1.41 | 0.48 – 4.12 | 0.43 |
|  | Harrel’s C = 0.75 | | | Harrel’s C = 0.76 | | | Not applicable | | |

vTTR stands for variant transthyretin amyloidosis.

**Figure S1. Receiving operating characteristic curve for the composite outcome and stroke volume index.**

The overall AUC was 0.60 while the best cut-off was 30.97 ml/m^2^, providing and AUC of 0.59 with a sensitivity of 58% and a specificity of 60%.

**Figure S2. Receiving operating characteristic curve for the composite outcome and stroke volume index.**

The overall AUC was 0.68 while the best cut-off was 40.5 mmHg, providing and AUC of 0.65 with a sensitivity of 61% and a specificity of 69%.

**Figure S3. Kaplan Meier Curves according to the SVi cut-off.**

SVi stands for Stroke Volume Index

**Figure S4. Kaplan Meier Curves according to sPAP cut-off.**

sPAP stands for Systolic Pulmonary Artery Pressure
